# Autumn phenology of tree species in China is associated more with climate than with spring phenology and phylogeny

**DOI:** 10.3389/fpls.2023.1040758

**Published:** 2023-01-19

**Authors:** Xinyue Gao, Junhu Dai, Zexing Tao, Khurram Shahzad, Huanjiong Wang

**Affiliations:** ^1^ Key Laboratory of Land Surface Pattern and Simulation, Institute of Geographic Sciences and Natural Resources Research (CAS), Beijing, China; ^2^ University of Chinese Academy of Sciences, Beijing, China; ^3^ China-Pakistan Joint Research Center on Earth Sciences, Chinese Academy of Sciences-Higher Education Commission of Pakistan, Islamabad, Pakistan

**Keywords:** leaf coloring date, climate change, spring phenology, phylogeny, China

## Abstract

Both biotic and abiotic factors restrict changes in autumn phenology, yet their effects remain ambiguous, which hinders the accurate prediction of phenology under future climate change. In this study, based on the phenological records of 135 tree species at ten sites in China during 1979–2018, we first investigated the effects of climatic factors (temperature, precipitation, insolation and wind speed) and spring phenology on interannual changes in leaf coloring date (LCD) with the partial correlation analysis, and assessed the relative importance of phylogeny and native climate to LCD differences among species by using multivariate regression and phylogenetic eigenvector regression approach. The results showed that the effects of climate factors on interannual changes in LCD were more significant than spring phenology. In general, temperature played a more important role in cold regions (e.g. the northeast region), while the control of insolation on LCD was stronger in the warmer and wetter regions (e.g. the north, east and southwest regions). In addition, the effects of precipitation and wind speed were more evident in arid regions (e.g. the northwest region). We also found considerable effects of both native climate and phylogeny on the LCD differences among species, despite the contribution of native climate being almost 2~5 times greater than that of the phylogeny. Our findings confirmed and quantified the combined effects of climate, spring phenology and phylogeny on the autumn phenology of plants, which could help better understand the driving factors and influencing mechanism of plant phenology and provide a reference for the calibration and optimization of phenological models.

## 1 Introduction

Plant phenology is the study of periodically recurring patterns of growth and development of plants during the year (Lieth, 1974; Richardson et al., 2013; Schwartz, 2013; Fu et al., 2018). Phenological variability affects ecosystem structure and functioning, which in turn controls strong vegetation feedbacks to climate systems (Menzel et al., 2006; Peñuelas et al., 2009; Klosterman et al., 2014; Piao et al., 2019b). Several studies have concluded that autumn phenology might have a more significant impact on the extent of growing season and changes in net ecosystem productivity than spring phenology (Fu et al., 2017; Zhang et al., 2018; Caparros-Santiago et al., 2021). Therefore, it is of great significance to explore the dynamics of autumn phenology to better understand the response of the ecosystem to climate change.

Over the past decades, a general trend toward delayed autumn phenology (e.g., the leaf coloring date, LCD) has been observed in temperate trees across the northern hemisphere (Vitasse et al., 2011; Ge et al., 2015; Jeong and Medvigy, 2015; Panchen et al., 2015; Yang et al., 2017; Piao et al., 2019a). However, the amplitude of such delaying trend varies among regions. Autumn phenology in China appears to have delayed (2.6 days per decade) more than in Europe (0.1 days per decade) during the period 1982-2011 (Ge et al., 2015; Piao et al., 2019a). Additionally, significant differences in trends of autumn phenology also exist among taxonomic groups. Rosbakh et al. (2021) observed a stronger delay of leaf senescence in herbs than in woody plants in Russia. Another study in China showed that the delaying trend of LCD for tree species was weaker than for shrub species (Dai et al., 2012). Given that the pattern of autumn phenology is highly complex, it is essential to investigate the response mechanisms of autumn phenology to environmental and biological factors.

At the interannual scale, a large number of studies have associated the variation in autumn phenology with the changes in several climatic and biological factors (**Figure S1**). Among climatic factors, temperature is generally regarded as the primary one of plant autumn phenology. The increase in summer/autumn temperature would delay the leaf senescence of temperate trees (Menzel et al., 2006; Delpierre et al., 2009; Vitasse et al., 2009; Črepinšek et al., 2012; Liu et al., 2016a; Liu et al., 2016b; Zohner et al., 2017). Two recent studies for temperate and subtropical vegetation further proposed that daytime maximum temperature and nighttime minimum temperature exhibited asymmetric effects on autumn phenology, which could be attributed to the contrasting influences of the daytime and nighttime temperature on the drought stress (Wu et al., 2018; Ren et al., 2021). Precipitation has also been proposed to be important to flowering phenology, especially in xeric systems, and an acceleration of leaf senescence was noted as a consequence of the reduced precipitation (Estrella and Menzel, 2006; Xie et al., 2015; Guo et al., 2021; Ren and Peichl, 2021). Additionally, the role of solar radiation, especially solar intensity, in triggering autumn phenology by affecting photosynthetic activities has also been reported in multiple studies (Calle et al., 2010; Liu et al., 2016a; Liu et al., 2016b). Moreover, evidence has been presented recently which suggests that the decline in winds in high-latitude areas could reduce evapotranspiration. Consequently, plants have more favorable growth conditions in late autumn and delay their timing of leaf senescence (Wu C et al., 2021). Apart from climatic factors, a few studies also observed earlier autumnal senescence due to earlier spring leaf out (Fu et al., 2014; Liu et al., 2016b; Yuan et al., 2018; Shen et al., 2020; Zani et al., 2020). Given that there are so many driving factors and the mechanism underlying the response of autumn phenology to climate change remains unclear, a thorough study of autumn phenology and its related controls is urgently needed.

Apart from the interannual scale, the variations in autumn phenology among species were also controlled by internal plant factors and climatic factors. Previous studies have shown that phylogeny might serve as the biological basis for specific phenological events of species (Rathcke and Lacey, 1985; Davies et al., 2013; CaraDonna and Inouye, 2015; Yang et al., 2021). The phylogenetic conservatism in the flowering and leaf-out date, which means that closely related species tend to have similar flowering and leaf-out dates because of their common ancestry (Rathcke and Lacey, 1985), has been confirmed in many studies (Davis et al., 2010; Davies et al., 2013; Staggemeier et al., 2015; Li et al., 2016; Cortés-Flores et al., 2017; Du et al., 2017). Apart from phylogeny, native climate, i.e., the climate in the native range of species, also affects plant phenology. Numerous studies have shown that species originating from higher latitudes leaf out earlier than species from lower latitudes when growing under identical conditions (Zohner and Renner, 2014; Zohner et al., 2016; Desnoues et al., 2017). However, it remains to be tested whether phylogeny affects autumn phenology and the relative importance of phylogeny and native climate on autumn phenology across species needs further investigation.

To address these issues, in this study, we sought to: (1) assess the long-term trends of LCD in different regions; (2) analyze the effects of climate factors (i.e., temperature, precipitation, insolation, wind speed) and first leaf date (FLD) on the interannual variation in LCD; and (3) explore the relative contributions of phylogeny and native climate to the variation in LCD among species. We hypothesized that though the main climatic drivers affecting LCD dynamics varied across different regions of China, the effects of climate drivers would be much more significant than FLD in all regions. Additionally, phylogeny would play an essential role in LCD variations. Still, its effect would be much weaker than native climate. Our results could help better identify the driving forces of autumn phenological changes and predict autumn phenology under future climate change.

## 2 Materials and Methods

### 2.1 Study area

**Figure 1:**
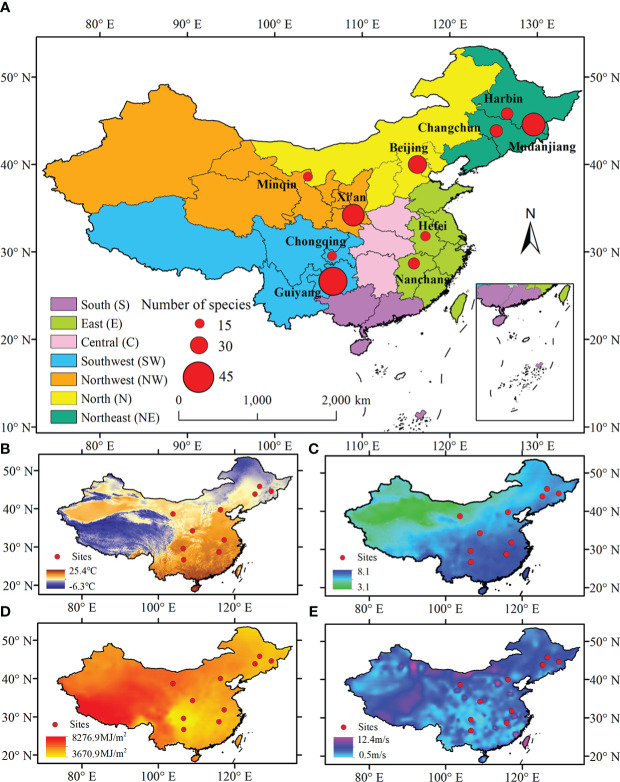
Spatial distributions of ten phenological observation sites in this study **(A)**. Spatial patterns of mean annual temperature **(B)**, mean annual precipitation (on a natural logarithm scale) **(C)**, mean annual solar radiation **(D)**, and mean annual wind speed **(E)** in China.

The diversity of bio-climate zones in China provides an excellent opportunity for identifying the effects of biotic and abiotic factors on LCD (**Figure 1**). This study selected ten sites of China Phenological Observation Network (CPON) (http://www.cpon.ac.cn/) with the most abundant autumn phenological observation records for analysis. To explore the spatial difference of the effects of various factors on LCD, we grouped the locations of the ten sites into different regions, so that the climate background of the sites within the same region was similar (**Table 1**, **Table S3**). The regions were divided according to provinces boundaries. Finally, the ten sites were assigned to five regions: the northeast China, the northwest China, the north China, the east China, and the southwest China. The plants in the sites in the same region experience similar climatic conditions. The mean annual temperature, mean annual precipitation, mean annual total solar radiation, and mean annual wind speed for these sites are 3.66 ~ 18.41°C, 164.27 ~ 1590.97 mm, 3762.19 ~ 6269.87 MJ/m^2^, and 1.38 ~ 2.98 m/s, respectively (**Table 1**).

**Table 1:** Climate background of each site in this study.

Region	Site	Mean annual temperature (°C)	Mean annual precipitation(mm)	Mean annual solar radiation (MJ/m^2^)	Mean annual wind speed (m/s)
Northeast	Harbin	5.15	523.93	4680.85	2.94
	Mudanjiang	3.66	569.90	4612.72	2.33
	Changchun	6.10	601.44	4925.32	2.98
North	Beijing	13.52	553.56	5260.33	2.08
Northwest	Minqin	9.51	164.27	6269.87	2.83
	Xi’an	10.95	498.01	5541.99	2.88
East	Hefei	16.50	1251.45	4797.16	2.58
	Nanchang	18.41	1590.97	4757.21	2.39
Southwest	Chongqing	17.69	1122.23	3762.19	1.38
	Guiyang	15.75	1102.34	3994.72	2.06

### 2.2 Phenology and climate data

The FLD and LCD observation records from 1979 to 2018 were obtained from CPON (**Figure S2**). According to the observation criteria (Wan and Liu, 1979), the FLD is defined as the date on which a fixed individual formed its first full leaf, and the LCD is defined as the date when the individual showed yellow leaves over 90% of its crown. Before analysis, an elementary data quality check was conducted to remove the LCD records corresponding to more than two times the median absolute deviation for each time series. Subsequently, we retained the species with at least 15 years of LCD and FLD data. Eventually, a total of 135 species at ten sites in five regions were chosen for analysis (**Table S1**).

Climate data, including daily maximum temperature (T_max_), minimum temperature (T_min_), precipitation (Pre), downward shortwave radiation (Ins), and wind speed (Win) from 1979 to 2018, were extracted from the China Meteorological Forcing Dataset (CMFD) (http://data.tpdc.ac.cn/), which is a fusion of remote-sensing products, reanalysis datasets, and *in situ* station data (Yang et al., 2010). The climate data at each site were extracted from the nearest pixel of gridded data (with a spatial resolution of 0.1°), and a statistical downscaling method was used to convert the data to the local scale (Hessami et al., 2008). We did not use observation data from meteorological stations mainly because of the lack of radiation data. In addition, some stations, such as Guiyang and Xi’an, have been relocated, resulting in discontinuous data which are difficult to calibrate. Based on the correlation analysis, we found that the climate data extracted from CMFD are highly correlated with the ground observation data (**Table S2**), showing that the climate data extracted from CMFD can effectively reflect the climate condition in each site, and hence could be used in this study.

The native climate for each species was determined by the following steps. Firstly, detailed information on native regions for each species (e.g., North America, South America, Europe and Asia) was obtained from Global Biodiversity Information Facility (GBIF, https://www.gbif.org), Flora of China (http://www.iplant.cn/), and other websites (http://linnaeus.nrm.se/flora/welcome.html; http://www.efloras.org/). Then, the occurrence records, including herbarium specimens and field observations from 1951 to 2021 within the native regions, were extracted from GBIF (**Figure S3**). The coordinate duplicates of the occurrence records for each species were removed. It was worth noting that the native regions of some species were well documented that their occurrence data could be extracted easily. However, for the species lacking detailed information on native regions, we used the continent with abundant occurrence records as their native regions (**Table S4**). Finally, for each species, we used the mean and range (minimum-maximum) of five climatic variables in all the occurrence points, including the mean annual temperature, the maximum temperature of the warmest month, the minimum temperature of the coldest month, mean annual precipitation and mean annual solar radiation, to express the native climate of the species. The climatic variables were acquired from the WorldClim dataset (https://worldclim.org/).

### 2.3 Methods

#### 2.3.1 Effects of climate drivers and spring phenology on LCD

Linear regression was applied to quantify the temporal variability of LCD during 1979–2018 for each species in each site. Next, the partial correlation analysis was used to investigate the effect of each factor (i.e., Tmax, Tmin, Pre, Ins, Win and spring phenology) on LCD with the effects of other factors removed. The relevant periods for the effects of each climate factor on LCD differ across species. The optimal length of the preseason of each climate factor was determined as the period for which the Pearson’s correlation coefficient between LCD and climate factor during 1, 2, 3,…, 150 days before the multi-year mean LCD was highest. The partial correlation could be calculated as:


(1) $$R_{\left(x,y|z\right)}=\frac{R_{xy}-R_{xz}\times R_{yz}}{\sqrt{1-R_{xz}^{2}}\times \sqrt{1-R_{yz}^{2}}}$$

Where *R*_(_ *_x,y|z_*_)_ is the partial correlation coefficient between variable x and variable y after controlling for the linear effect of variable z; *R_xy_*, *R_xz_* and *R_yz_* are correlation coefficients between variable x and variable y, variable x and variable z, and variable y and variable z, respectively. *R*_(_ *_x,y|z_*_)_>0 means a positive correlation between variable *x* and variable *y*, and *R*_(_ *_x,y|z_*_)_<0 means a negative correlation between them.

For each region and each variable, the average absolute value of partial correlation coefficient (|R|) of all species was calculated. A higher |R| means a more crucial effect of the variable altering LCD.

#### 2.3.2 Effects of phylogeny and native climate on LCD

We investigated the effects of phylogeny and native climate on the interspecific variation in LCD through the following three steps.

*Step 1: Construction of phylogenetic trees*. The scientific names of the studied species were first obtained from Plant List (www.theplantlist.org). Next, we used a mega phylogeny provided by the Open Tree of Life Version 9.1, which included 74,533 taxa and a backbone with all extant vascular plant families, to generate a phylogeny for all studied species in each site. The phylogeny tree of the studied species was constructed by the R package V. PhyloMaker (Jin and Qian, 2019; Qian and Jin, 2020).

*Step 2: Test of phylogenetic signal in LCD*. Blomberg’s K is a widely used phylogenetic signal method. It indicates the strength of the tendency of closely related species to have similar phenological traits (Blomberg et al., 2003). Here, we used Blomberg’s K to quantify the strength of phylogenetic signals in LCD (multi-year average) to investigate whether LCD was affected by phylogeny. Blomberg’s K compares the observed distribution of tip data to expectations derived from a Brownian motion model of evolution (Bhaskar et al., 2016). *K* = 1 indicates that LCD conforms to Brownian motion evolution, while *K* = 0 indicates that the LCD is independent of the phylogenetic relationships (Blomberg et al., 2003). The *p* value was also obtained with 1000 interactions in the calculation of *K* to detect whether the observed values differed significantly from a randomized arrangement. If *p* > 0.05, we would reject the phylogenetic conservatism hypothesis, meaning that the LCD was not controlled by phylogeny (Blomberg et al., 2003). Blomberg’s K was calculated by the package phytools (Revell, 2011).

*Step 3: Quantifying the relative contributions of phylogenetic and native climate in determining the interspecific variation in LCD*. We used multivariate phylogenetic eigenvector regressions analysis (PVR) to identify the relative contributions of native climate and phylogeny to the interspecific variation in LCD (Diniz-Filho et al., 1998; Desdevises et al., 2003). To obtain native climatic factors, we first modeled LCD as a function of the ten native climatic variables (i.e., the mean and range of five native climatic variables) and used stepwise regression to select the best set of native climatic variables predicting the LCD. Phylogenetic eigenvectors were acquired based on the pairwise phylogenetic distance matrix between species carried out with principal coordinate analysis (PCoA). Each eigenvector obtained from PCoA represents a portion of the phylogenetic variance in the evolutionary history of these species. We used Moran’s I approach (Diniz-Filho et al., 2012) to determine the eigenvectors subsets that minimize autocorrelation in the residuals of phylogenetic regressions by PVR package in R. These eigenvectors were used as phylogenetic factors in subsequent regressions.

To untangle the exclusive influence attributed to phylogeny (*P_1_*), to native climate (*P_2_*) and shared influence by both (*P_3_*), we combined the native climate and phylogeny factors described above in three multivariate regressions, with a same dependent variable of multiyear mean LCD, and different independent variables. Three adjusted R^2^ (with units of %) attributed to *P_1_*, *P_2_* and *P_3_* were calculated as:


(1) $$P_{1}=R_{adj3}^{2}-R_{adj1}^{2}$$

(2) $$P_{2}=R_{adj3}^{2}-R_{adj2}^{2}$$

(3) $$P_{3}=R_{adj2}^{2}-(R_{adj3}^{2}-R_{adj1}^{2})$$

$R_{adj1}^{2}$ is the adjusted R^2^ obtained by modeling LCD as a function of the native climatic variables; $R_{adj2}^{2}$ is the adjusted R^2^ obtained by modeling LCD as a function of phylogenetic variables; and $R_{adj3}^{2}$ is the adjusted R^2^ obtained by modeling LCD as a function of the native climatic variables and phylogenetic variables.

The *P_1_*, *P_2_* and *P_3_* were first obtained in each site. The native climate and phylogeny contributions for each region were calculated as the average *P_1_*, *P_2_* and *P_3_* values for all regional sites.

## 3 Results

### 3.1 Phenological trends of LCD

**Figure 2:**
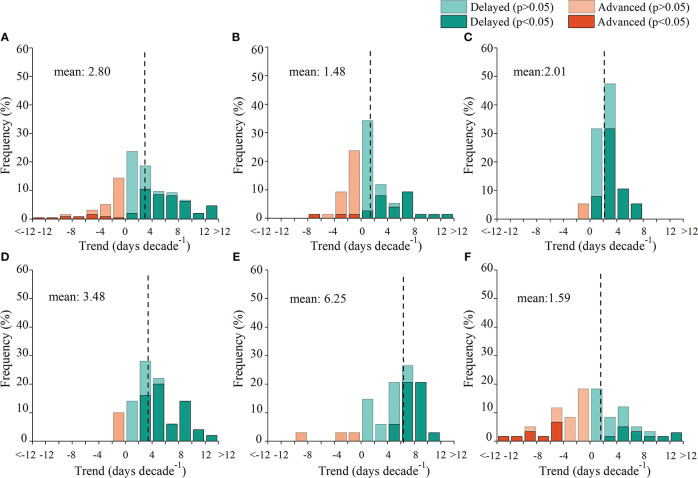
Frequency distribution of the trend in leaf coloring date (LCD) for all regions **(A)**, the northeast **(B)**, north **(C)**, northwest **(D)**, east **(E)**, and southwest **(F)** region during 1979–2018. The vertical lines represent the mean trends of LCD.

A total of 73.1% of the species showed delayed LCD during 1979–2018 (**Figure 2**), of which 40.3% were significant (*p<* 0.05). Overall, phenological records indicated that LCD had delayed an average of 2.80 days per decade for the last four decades. Additionally, the magnitude of the trend of LCD varied among regions. The mean delaying trend of LCD was strongest in the east region (6.25 days/decade) and was weakest in the northeast region (1.48 days/decade).

### 3.2 The effects of climate drivers and FLD on LCD

**Figure 3:**
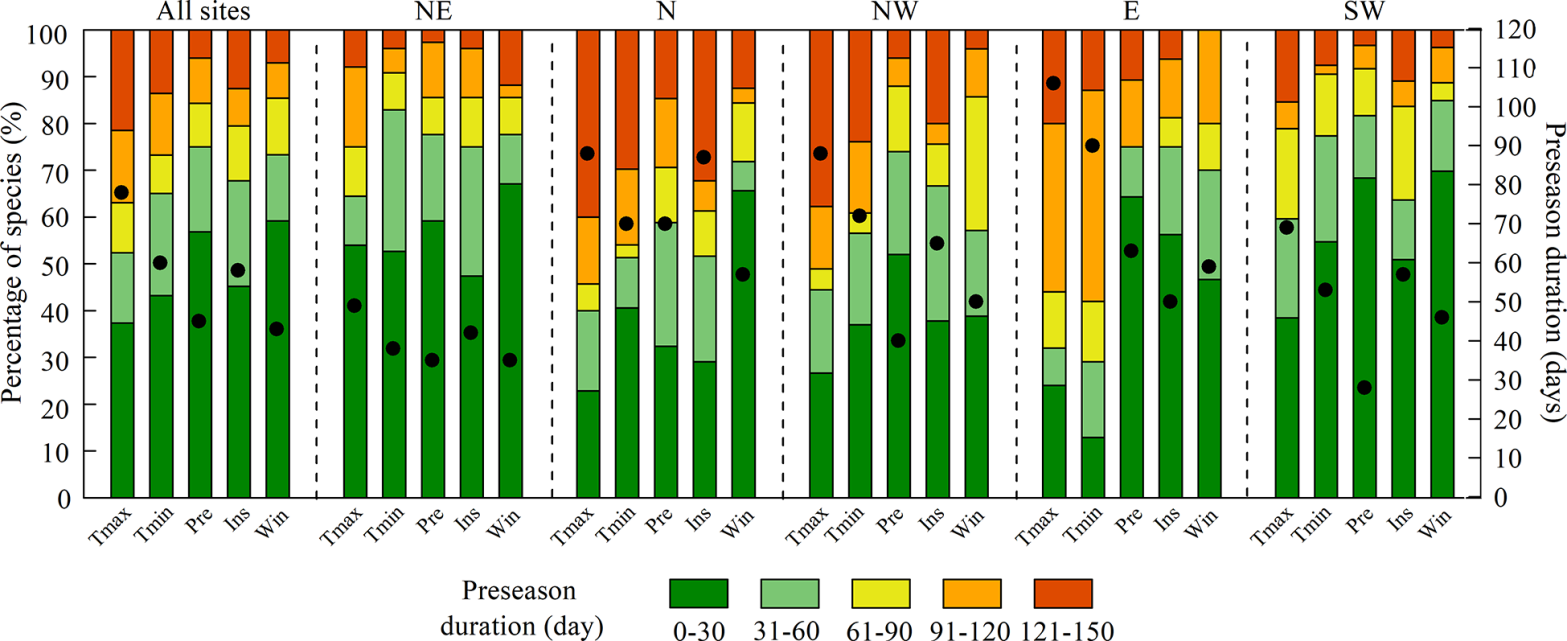
Preseason durations for maximum temperature (T_max_), minimum temperature (T_min_), precipitation (Pre), shortwave radiation (Ins), and wind speed (Win) for all sites and each region. NE: northeast; N: north; NW: northwest; E: east; SW: southwest. The black dots indicate the mean preseason duration (right y-axis) of all species.

The mean preseason durations of T_max_, T_min_, Pre, Ins, and Win across all sites were 78, 60, 45, 58, and 43 days, respectively (**Figure 3**). That is, the LCD was primarily influenced by climate factors in two or three months before LCD. The preseason duration of T_max_ was longer than that of the other climate factors in all regions, indicating an earlier response of LCD to preseason T_max_.

**Figure 4:**
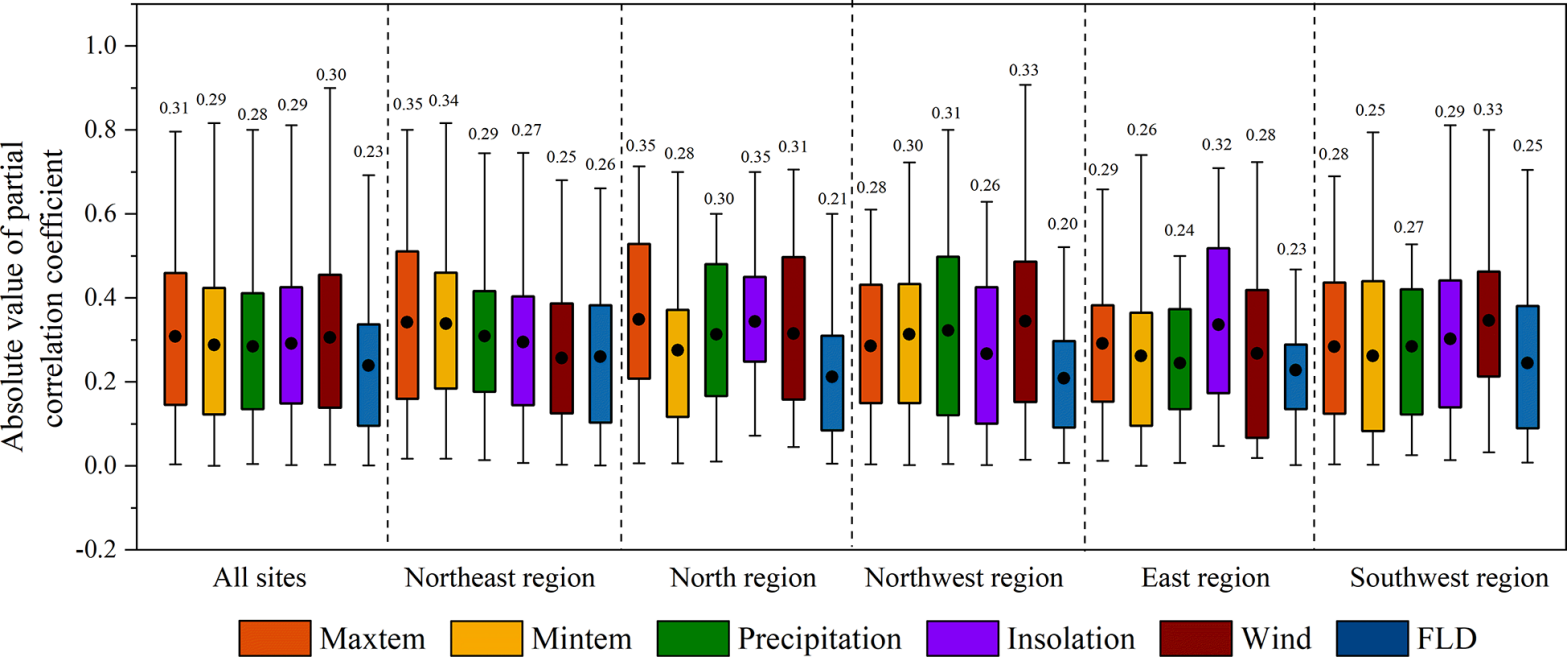
The absolute values of the partial correlation coefficients between LCD and climatic variables, first leaf date (FLD) in different regions. The black dots and the numbers indicate the mean value of the coefficients of all species.

**Figure 5:**
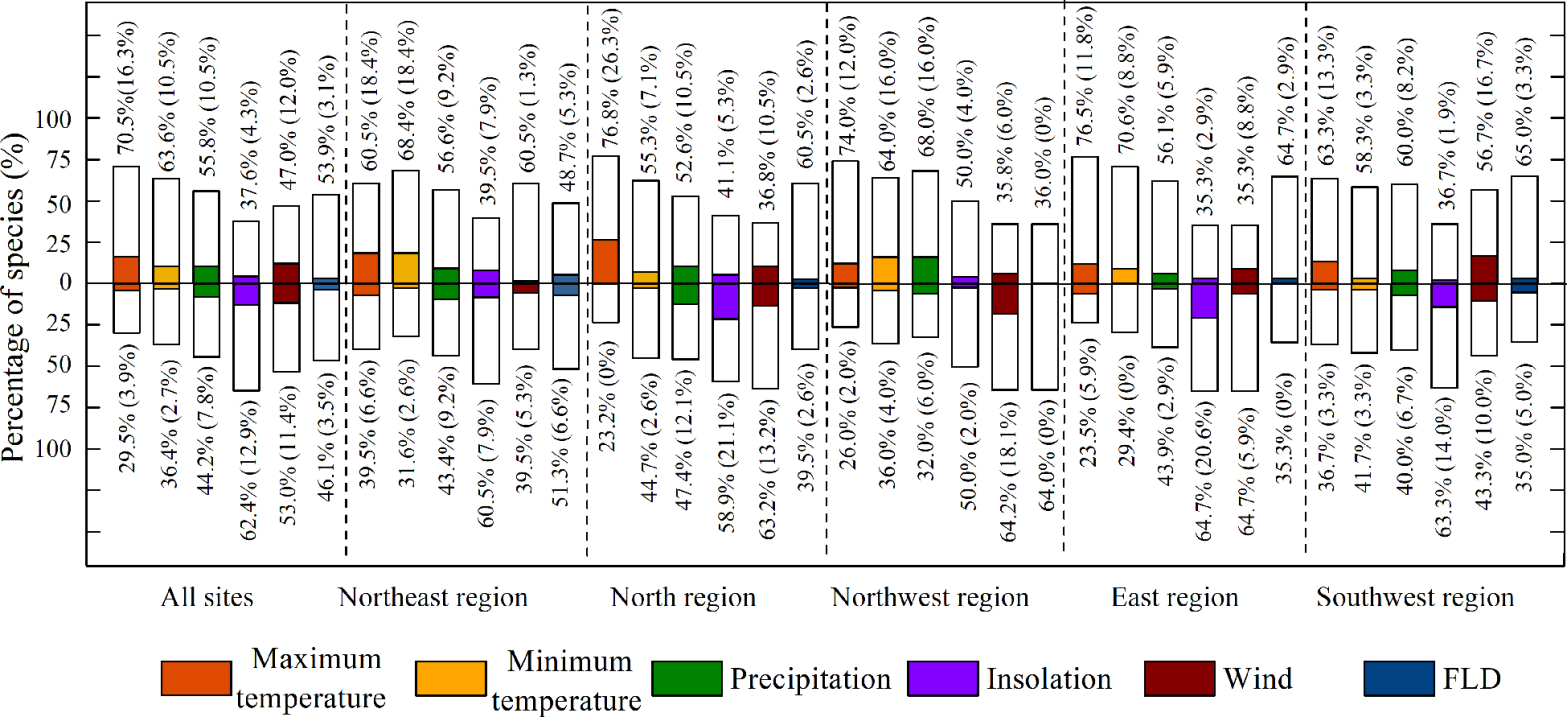
Partial correlations between LCD and five climatic variables, first leaf date (FLD) in different regions. Bars above zero represent the percentage of positive correlations and those under zero show percentages of negative correlations. The colored bars and numbers in brackets indicate the percentage of significant correlations at *p*<0.05.

In general, LCD was more correlated with climate factors than with FLD, as the absolute values of the partial correlation coefficients (|*R|*) between LCD and climate factors (0.28~0.31) were larger than that between LCD and FLD (0.23) (**Figure 4**). The effects of climate divers on LCD were different among regions (**Figure 5**). T_max_ and T_min_ were mainly positively correlated with LCD in all regions. This positive correlation was strong in the northeast region (|*R|*>0.34). Strong positive correlations between LCD and precipitation were found in the northwest region. However, the effect of precipitation in other regions was ambiguous, as the proportions of negative and positive partial correlation coefficients were almost similar. Compared with other regions, LCD showed a larger |*R|* with insolation in the north, east and southwest regions, where an increase in insolation mainly led to an advance in LCD. Moreover, the correlation between LCD and wind speed was strong in the northwest and southwest regions.

Nevertheless, interestingly, most species (64.2%) showed negative correlations between LCD and wind speed in the northwest region (significant: 18.1%), while 56.7% of the species showed positive correlations between LCD and winds (significant: 16.7%) in the southwest area.

### 3.3 The phylogenetic signal of LCD

In general, the Blomberg’s K of LCD was less than 1, indicating that LCD was less similar to what would be expected under Brownian motion (**Table 2**). The strength of the phylogenetic signal in LCD was weakest in the northeast region (*K*=0.15), while it was strongest in the northwest region (*K*=0.56). However, LCD was not phylogenetically conserved in all sites as none of the signals were significant (except for Minqin).

**Table 2:** The phylogenetic signal of LCD.

Region	Average Blomberg’s K	Site	*K*-Value	*p -*Value
Northeast	0.15	Harbin	0.12	0.38
		Mudanjiang	0.06	0.75
		Changchun	0.28	0.61
North	0.29	Beijing	0.29	0.08
Northwest	0.56	Minqin	0.79	0.03
		Xi’an	0.33	0.55
East	0.40	Hefei	0.35	0.86
		Nanchang	0.45	0.52
Southwest	0.29	Chongqing	0.47	0.46
		Guiyang	0.11	0.83

### 3.4 Relative contributions of phylogeny and native climate to species-specific variation in LCD

**Figure 6:**
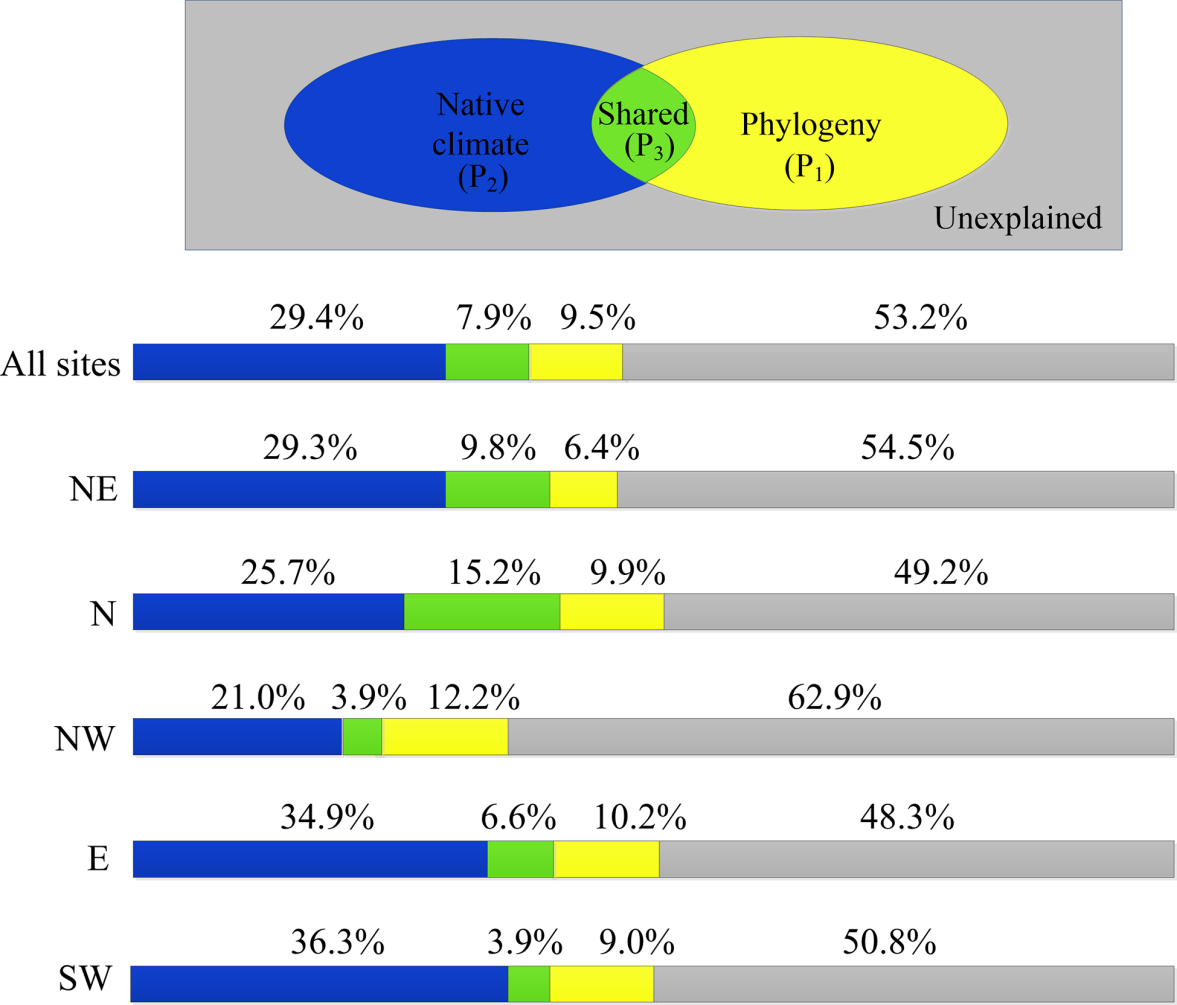
Fractions of species-level variations (adjusted R^2^) in LCD explained by native climate (blue), phylogeny (yellow) and both (green). NE: northeast; N: north; NW: northwest; E: east; SW: southwest.

Native climate and phylogeny explained on average 46.8% of species-specific shifts in LCD for all regions (**Figure 6**). The contribution of native climate to the variations in LCD across species was 2 to 5 times greater than that of the phylogeny. That is, native climate better explained LCD variation among species than phylogeny. At the regional level, the explanatory power of phylogeny was greatest in the northwest region (12.2%) but smallest in the northeast region (6.4%). For native climate, it was more dominant in warmer and wetter regions (the east and southwest regions) but less so in arid regions (northwest region).

## 4 Discussion

### 4.1 Temporal pattern of LCD

We noticed a delayed LCD from 1979–2018 in study areas, which confirms the findings of the delaying trend in previous studies for temperate trees across the Northern Hemisphere (Vitasse et al., 2011; Panchen et al., 2015; Ge et al., 2015; Piao et al., 2019a). However, the amplitude of delaying trend of LCD in our study (2.8 days per decade) differed from that in temperate China (1.2 days per decade) (Liu et al., 2016a) and Eastern USA (4.1 days per decade) (Dragoni and Rahman, 2012) based on the remote sensing data, and in Switzerland (1.5 days per decade) (Bigler and Vitasse, 2021) based on *in-situ* observation records. The discrepancies could be attributed to the differences in the phenology data sources and study region. First, the phenology obtained by ground observation is at the individual scale, which commonly refers to the phenology of dominant species. However, the phenology extracted from remote sensing data is at the community scale, which majorly reflects the phenology of the vegetation canopy. In addition, plants may exhibit different phenology in diverse climate conditions due to their adaptation and plasticity (Zohner and Renner, 2014). For example, the climate in high elevations in Switzerland is more severe than in our studied areas (Bigler and Vitasse, 2021), so the plants in Switzerland might have adapted strongly to the drought and cold climate. Delay in the LCD of plants means an extension in the length of the growing season, which can increase plants’ carbon assimilation and raise vegetation productivity (Richardson et al., 2013; Wu L et al., 2021). For example, a delay of 3 d yr^-1^ in leaf senescence paralleled an increase in net ecosystemic productivity of 5 g C m^-2^ yr^-1^ in late summer for deciduous forests (Dragoni and Rahman, 2012). Changes in the phenology of leaf senescence could also impact ecosystem nutrient cycling and further the functions and sustainability of the terrestrial ecosystem (Estiarte and Peñuelas, 2015).

### 4.2 Effects of climate factors and spring phenology on LCD

Our results showed that the increase of T_max_ and T_min_ in late summer and autumn delayed LCD in all regions, which is consistent with previous studies which also reported a delay in autumn phenology due to the increased temperature (Vitasse et al., 2009; Ge et al., 2015; Liu et al., 2016a; Liu et al., 2016b; Zani et al., 2020). The positive correlation between LCD and temperature may be explained by the higher temperatures enhancing the activities of photosynthetic enzymes (Shi et al., 2014) and postponing the degradation of chlorophyll (Fracheboud et al., 2009; Yang et al., 2017). In addition, we found that T_max_ and T_min_ had strong effects on LCD in the cold region (i.e., the northeast region), likely because the temperature is a limiting factor for plant growth in the northeast region (where the mean temperature in summer and autumn is below 20°C). A higher daytime temperature could substantially promote photosynthesis (Liu et al., 2016a), and an increased nighttime temperature could significantly mitigate soil frosting and further promote root absorption under heavy cold stress (Pangtey et al., 1990; Wan et al., 2009).

Consistent with other studies (Liu et al., 2016a; Liu et al., 2016b; Guo et al., 2021; Ren et al., 2021), we also found that increased precipitation significantly delayed LCD in drier regions. The explanation might be that increasing precipitation could alleviate drought stress in dry regions and reduce plant mortality (Estiarte and Peñuelas, 2015; Bertani et al., 2017).

The effect of insolation was more evident in the warmer and wetter areas, i.e., the north, east and southwest regions, likely because the temperature and precipitation in these regions were more favorable than other regions with respect to supporting the efficiency of photosynthesis in using light radiation (Sofo et al., 2009; Guo et al., 2021). The carbon-sink limitation of plants may explain the negative effect of insolation on LCD in these areas. Under higher insolation, the carbon capture in plants is elevated, which prohibits the capacity of photosynthesis and acts as a self-regulatory mechanism to constrain the productive season length (Zani et al., 2020). Consequently, higher insolation could lead to an earlier LCD. Although some studies have shown that increased insolation would retard the accumulation of abscisic acid and subsequently slow down the rate of leaf senescence (Liu et al., 2016a; Liu et al., 2016b), we speculate this delaying effect may be weaker than carbon-sink limitation.

We found that increased wind speed would cause an earlier LCD in the northwest region but a later LCD in the southwest region. In the northwest region, strong winds may lead to an increase in evapotranspiration and a reduction in soil water, resulting in an earlier LCD (Wu et al., 2021). In the southwest region, ecological processes may be more important than physiological effects of water stress in explaining the response of LCD to winds. Plants could adapt to specific environments and develop several acclimation approaches with attributes of reorientation and reconfiguration subject to wind variations (Harder et al., 2004). They can therefore survive if the winds are not too strong and can even grow better where water and nutrients supplies are sufficient because their adaptations to winds may lead to intrinsic differences in plants’ timing of foliar senescence that are optimized to maximize carbon gain and minimize the loss of water (Caldwell, 1970; Harder et al., 2004; Gardiner et al., 2016; Wu et al., 2018). Thus, an increase in wind speed may result in an extension of the growing season, i.e., a later LCD. Interestingly, we found that wind speed was more crucial than other climate factors in the northwest and southwest regions. The reason might be that plants in the northwest areas are mainly threatened by water shortage, which is closely related to wind-reduced evapotranspiration. In the southwest region, hydrothermal conditions are suitable for plants. Thus plant autumn phenology is less sensitive to changes in temperature and precipitation. On the contrary, the wind here is relatively weak (mean annual wind speed: 1~2 m s^-1^). The increase in the wind over the past decade (ca. 0.23m s^-1^ decade^-1^) may significantly alter the LCD of plants. Consequently, wind speed is more crucial than other climate factors in the regions. Overall, the effect of winds on autumn phenology received less attention. Further experimental studies should be carried out to reveal the response mechanism of phenology to wind in different areas.

Similar to previous studies (Fu et al., 2014; Zani et al., 2020), we found that an earlier spring phenology was followed by an earlier LCD in the north and east regions. There are two possible reasons. The first reason is that the advancement of spring onset would facilitate vegetation growth and consume more moisture in the earlier stage, which may cause summer droughts, productivity reduction, and earlier leaf senescence at later phases (Keenan and Richardson, 2015). The second reason is that non-structural carbohydrates accumulate faster after early spring phenology, which may result in an earlier peak in autumn carbon contents, and therefore, the LCD would be advanced accordingly (Buermann et al., 2013; Fu et al., 2014). In addition, we also observed a delaying effect of earlier spring phenology on LCD in the northwest region, which is similar to another study showing a negative correlation between spring phenology and LCD in the deciduous forest (Liu et al., 2016b). Unfortunately, current understandings are not able to fully explain this correlation, and further research in the spring phenology effects on LCD in different areas should be more considered. It is worth to be noted that the control of FLD was much weaker than that of climatic factors in all regions, implying that the climatic factors should be considered more than spring phenology when predicting autumn phenology.

### 4.3 Effects of phylogeny and native climate on LCD

Previous studies have suggested phylogenetic conservatism in spring phenology but not in autumn phenology (Davies et al., 2013; Panchen et al., 2015; Du et al., 2017). In this study, we also found that the LCD of the species was poorly phylogenetically conserved. Regarding the strength of the phylogenetic signal, we detected the weakest phylogenetic signal of LCD in the northeast region, presumably due to the stronger environmental filtering effect (Pau et al., 2011). Plants must balance the need to prolong the growing season and the benefits of minimizing damage to plant organs. In the northeast region, where the temperature is highly unfavorable, plants might adjust the phenology of leaf senescence to a specific time in a narrow window with favorable growth conditions (Cavender-Bares et al., 2009; Lessard-Therrien et al., 2014). In other words, the strong abiotic selection pressure overrides the shared evolutionary history, resulting in the close autumn phenology of all species in the northeast region, thus presenting a weak phylogenetic signal (Lessard-Therrien et al., 2014). The explanation for the strongest signal in the northwest region may be that the flora in the western region shows more recent genetic divergence (divergence times of 15.29–18.86 Mya) compared with those in other areas (divergence times of 22.04–25.39 Mya) (Lu et al., 2018). Consequently, the phenology traits of plant species in the northwest region are more similar to those of their ancestors, thus showing a strong phylogenetic signal.

In general, we found that the effect of native climate on autumn phenology was more significant than that of phylogeny, which, to our knowledge, has seldomly been reported in other studies. Regarding the regional difference, the finding that native climate was more dominant in warmer regions (e.g., the east and southwest regions) might associate with less climate stress on species’ evolution. Therefore, species could retain the adaptation strategy to their native climate other than the climate conditions in studied sites. The proportion of the LCD variation among species that was neither explained by native climate nor by phylogeny may be related to biological traits such as growth habits, woody anatomical structures (Dai et al., 2012; Panchen et al., 2015; Cortés-Flores et al., 2018), and short-term weather conditions such as frost or rain (Nagy et al., 2013). Future studies can compare the phenological patterns among different communities for better understanding the relative importance of abiotic, biotic, and evolutionary factors to plant phenology.

### 4.4 Limitations of the study

There are some uncertainties in this study. Due to the lack of more detailed information on the native range of species, we grouped the native ranges into several continents. This method of categorizing the native regions is relatively coarse and might result in uncertainty in detecting the native climate of species. Based on the available data, we could only refer to the approach of Zohner and Renner (2014), using continents with the most abundant occurrence records to represent native areas. More detailed information about the native range of species and more accurate classification of native regions through field survey and document compilation would be favored for future studies.

## 5 Conclusions

This study investigated the effects of biotic and abiotic factors on autumn phenology in China. The results revealed that the LCD was delayed by 2.80 days/decade on average for the last four decades. At the interannual scale, the effects of climate factors on the leaf coloring dates were greater than that of the first leaf date. Temperature played a more important role in cold regions (e.g. northeast region), while the control of insolation on LCD was stronger in the warmer and wetter regions (e.g. the north, east and southwest regions). In addition, the effects of precipitation and wind speed were more evident in arid regions (e.g. northwest region). At the species level, native climate played a more vital role than phylogeny in determining LCD differences among species. The contribution of native climate was almost 2~5 times greater than that of phylogeny. Overall, the results highlight the more significant role of climate on autumn phenology than biotic factors (i.e., spring phenology and phylogeny). More in-depth studies on the interannual change and interspecific variation in LCD are urgently needed for a better understanding of the interactions between plant phenology and biotic and environmental controls.

## Data availability statement

The original contributions presented in the study are included in the article/**Supplementary Material**. Further inquiries can be directed to the corresponding author.

## Author contributions

JD and ZT conceived the idea. XG analyzed the data and wrote the draft. ZT, KS and HW revised the manuscript. All authors contributed to the article and approved the submitted version.

## References

[B1] Bertani G. Wagner F. H. Anderson L. O. Aragao L. (2017). Chlorophyll fluorescence data reveals climate-related photosynthesis seasonality in Amazonian forests. Remote Sens. 9 (12), 1275. doi: 10.3390/rs9121275

[B2] Bhaskar R. Porder S. Balvanera P. Edwards E. J. (2016). Ecological and evolutionary variation in community nitrogen use traits during tropical dry forest secondary succession. Ecology 97 (5), 1194–1206. doi: 10.1890/15-1162.1 27349096

[B3] Bigler C. Vitasse Y. (2021). Premature leaf discoloration of European deciduous trees is caused by drought and heat in late spring and cold spells in early fall. Agric. For. Meteorol. 307, 108492. doi: 10.1016/j.agrformet.2021.108492

[B4] Blomberg S. P. Garland T. Ives A. R. (2003). Testing for phylogenetic signal in comparative data: Behavioral traits are more labile. Evolution 57 (4), 717–745. doi: 10.1111/j.0014-3820.2003.tb00285.x 12778543

[B5] Buermann W. Bikash P. Jung M. Burn D. Reichstein M. (2013). Earlier springs decrease peak summer productivity in north American boreal forests. Envioromental Res. Lett. 8 (2), 024027. doi: 10.1088/1748-9326/8/2/024027

[B6] Caldwell M. M. (1970). Plant gas exchange at high wind speeds. Plant Physiol. 46 (4), 535–537. doi: 10.1104/pp.46.4.535 16657501 PMC396631

[B7] Calle Z. Schlumpberger B. O. Piedrahita L. Leftin A. Hammer S. A. Tye A.. (2010). Seasonal variation in daily insolation induces synchronous bud break and flowering in the tropics. Trees 24, 865–877. doi: 10.1007/s00468-010-0456-3

[B8] Caparros-Santiago J. A. Rodriguez-Galiano V. Dash J. (2021). Land surface phenology as indicator of global terrestrial ecosystem dynamics: A systematic review. ISPRS J. Photogrammetry Remote Sens. 171, 330–347. doi: 10.1016/j.isprsjprs.2020.11.019

[B9] CaraDonna P. J. Inouye D. W. (2015). Phenological responses to climate change do not exhibit phylogenetic signal in a subalpine plant community. Ecology 96 (2), 355–361. doi: 10.1890/14-1536.1 26240857

[B10] Cavender-Bares J. Kozak K. H. Fine P. Kembel S. W. (2009). The merging of community ecology and phylogenetic biology. Ecol. Lett. 12 (7), 693–715. doi: 10.1111/j.1461-0248.2009.01314.x 19473217

[B11] Cortés-Flores J. Cornejo-Tenorio G. Urrea-Galeano L. A. Andresen E. González-Rodríguez A. Ibarra-Manríquez G. (2018). Phylogeny, fruit traits, and ecological correlates of fruiting phenology in a Neotropical dry forest. Oecologia 189 (1), 159–169. doi: 10.1007/s00442-018-4295-z 30411150

[B12] Cortés-Flores J. Hernández-Esquivel K. González-Rodríguez A. Ibarra-Manríquez G. (2017). Flowering phenology, growth forms, and pollination syndromes in tropical dry forest species: Influence of phylogeny and abiotic factors. Am. J. Bot. 104 (1), 39–49. doi: 10.3732/ajb.1600305 28031168

[B13] Črepinšek Z. Štampar F. Kajfež-Bogataj L. Solar A. (2012). The response of corylus avellana l. phenology to rising temperature in north-eastern Slovenia. Int. J. Biometeorol. 56 (4), 681–694. doi: 10.1007/s00484-011-0469-7 21786017

[B14] Dai J. Wang H. Ge Q. (2012). Multiple phenological responses to climate change among 42 plant species in xi’an, China. Int. J. Biometeorol. 57 (5), 749–758. doi: 10.1007/s00484-012-0602-2 23114575

[B15] Davies T. J. Wolkovich E. M. Kraft N. J. B. Salamin N. Allen J. M. Ault T. R.. (2013). Phylogenetic conservatism in plant phenology. J. Ecol. 101 (6), 1520–1530. doi: 10.1111/1365-2745.12154

[B16] Davis C. C. Willis C. G. Primack R. B. Miller-Rushing A. J. (2010). The importance of phylogeny to the study of phenological response to global climate change. Philos. Trans. R. Soc. B: Biol. Sci. 365 (1555), 3201–3213. doi: 10.1098/rstb.2010.0130 PMC2981945 20819813

[B17] Delpierre N. Dufrêne E. Soudani K. Ulrich E. Cecchini S. Boé J.. (2009). Modelling interannual and spatial variability of leaf senescence for three deciduous tree species in France. Agric. For. Meteorol. 149 (6-7), 938–948. doi: 10.1016/j.agrformet.2008.11.014

[B18] Desdevises Y. Legendre P. Morand A. S. (2003). Quantifying phylogenetically structured environmental variation. Evolution 57 (11), 2647–2652. doi: 10.1111/j.0014-3820.2003.tb01508.x 14686540

[B19] Desnoues E. Ferreira, de Carvalho J. Zohner C. M. Crowther T. W. (2017). The relative roles of local climate adaptation and phylogeny in determining leaf-out timing of temperate tree species. For. Ecosyst. 4 (1), 1–7. doi: 10.1186/s40663-017-0113-z

[B20] Diniz-Filho J. A. F. Bini L. M. Rangel T. F. Morales-Castilla I. Olalla-Tarraga M.Á. Rodriguez M.Á.. (2012). On the selection of phylogenetic eigenvectors for ecological analyses. Ecography 35 (3), 239–249. doi: 10.1111/j.1600-0587.2011.06949.x

[B21] Diniz‐Filho J. A. F. Sant'Ana C. E. R. D. Bini L. M. (1998). An eigenvector method for estimating phylogenetic inertia. Evolution 52 (5), 1247–1262. doi: 10.1111/j.1558-5646.1998.tb02006.x 28565378

[B22] Dragoni D. Rahman A. F. (2012). Trends in fall phenology across the deciduous forests of the Eastern USA. Agric. For. Meteorol. 157, 96–105. doi: 10.1016/j.agrformet.2012.01.019

[B23] Du Y. Chen J. Willis C. G. Zhou Z. Liu T. Dai W.. (2017). Phylogenetic conservatism and trait correlates of spring phenological responses to climate change in northeast China. Ecol. Evol. 7 (17), 6747–6757. doi: 10.1002/ece3.3207 28904756 PMC5587463

[B24] Estiarte M. Peñuelas J. (2015). Alteration of the phenology of leaf senescence and fall in winter deciduous species by climate change: effects on nutrient proficiency. Global Change Biol. 21 (3), 1005–1017. doi: 10.1111/gcb.12804 25384459

[B25] Estrella N. Menzel A. (2006). Responses of leaf colouring in four deciduous tree species to climate and weather in Germany. Climate Res. 32 (3), 253–267. doi: 10.3354/cr032253

[B26] Fracheboud Y. Luquez V. Bjorken L. Sjodin A. Tuominen H. Jansson S. (2009). The control of autumn senescence in European aspen. Plant Physiol. 149 (4), 1982–1991. doi: 10.1104/pp.108.133249 19201914 PMC2663763

[B27] Fu Y. Campioli M. Vitasse Y. Boeck H. D. Van D. Abdelgawad H.. (2014). Variation in leaf flushing date influences autumnal senescence and next year’s flushing date in two temperate tree species. Proc. Natl. Acad. ences 111 (20), 7355–7360. doi: 10.1073/pnas.1321727111 PMC4034254 24799708

[B28] Fu Y. He H. S. Zhao J. Larsen D. R. Zhang H. Sunde M. G.. (2018). Climate and spring phenology effects on autumn phenology in the greater khingan mountains, northeastern China. Remote Sens. 10 (3), 449. doi: 10.3390/rs10030449

[B29] Fu Y. Piao S. Delpierre N. Hao F. Hänninen H. Liu Y.. (2017). Larger temperature response of autumn leaf senescence than spring leaf-out phenology. Global Change Biol. 24 (5), 2159–2168. doi: 10.1111/gcb.14021 29245174

[B30] Gardiner B. Berry P. Moulia B. (2016). Wind impacts on plant growth, mechanics and damage. Plant Sci. 245, 94–118. doi: 10.1016/j.plantsci.2016.01.006 26940495

[B31] Ge Q. Wang H. Rutishauser T. Dai J. (2015). Phenological response to climate change in China: a meta-analysis. Global Change Biol. 21 (1), 265–274. doi: 10.1111/gcb.12648 24895088

[B32] Guo M. Wu C. Peng J. Lu L. Li S. (2021). Identifying contributions of climatic and atmospheric changes to autumn phenology over mid-high latitudes of northern hemisphere. Global Planetary Change 197 (6237), 103396. doi: 10.1016/j.gloplacha.2020.103396

[B33] Harder D. L. Speck O. Hurd C. L. Speck T. (2004). Reconfiguration as a prerequisite for survival in highly unstable flow-dominated habitats. J. Plant Growth Regul. 23 (2), 98–107. doi: 10.1007/s00344-004-0043-1

[B34] Hessami M. Gachon P. Ouarda T. St-Hilaire A. (2008). Automated regression-based statistical downscaling tool. Environ. Model. Softw. 23(6), 813–834. doi: 10.1016/j.envsoft.2007.10.004

[B35] Jeong S. J. Medvigy D. (2015). Macroscale prediction of autumn leaf coloration throughout the continental united states. Global Ecol. Biogeogr. 23 (11), 1245–1254. doi: 10.1111/geb.12206

[B36] Jin Y. Qian H. (2019). V.PhyloMaker: an r package that can generate very large phylogenies for vascular plants. Ecography 42 (8), 1353–1359. doi: 10.1111/ecog.04434 PMC9363651 35967255

[B37] Keenan T. F. Richardson A. D. (2015). The timing of autumn senescence is affected by the timing of spring phenology: implications for predictive models. Global Change Biol. 21, 2634–2641. doi: 10.1111/gcb.12890 25662890

[B38] Klosterman S. T. Hufkens K. Gray J. M. Melaas E. Sonnentag O. Lavine I.. (2014). Evaluating remote sensing of deciduous forest phenology at multiple spatial scales using PhenoCam imagery. Biogeosciences 11 (16), 4305–4320. doi: 10.5194/bg-11-4305-2014

[B39] Lessard-Therrien M. Davies T. J. Bolmgren K. (2014). A phylogenetic comparative study of flowering phenology along an elevational gradient in the Canadian subarctic. Int. J. Biometeorol. 58 (4), 455–462. doi: 10.1007/s00484-013-0672-9 23686022

[B40] Lieth H. (1974). Phenology and seasonality modeling (New York, NY: Springer-Verlag Berlin Heidelberg).

[B41] Li L. Li Z. Cadotte M. W. Jia P. Chen G. Jin L. S.. (2016). Phylogenetic conservatism and climate factors shape flowering phenology in alpine meadows. Oecologia 182 (2), 419–428. doi: 10.1007/s00442-016-3666-6 27351544

[B42] Liu Q. Fu Y. Zeng Z. Huang M. T. Li X. Piao S. (2016a). Temperature, precipitation, and insolation effects on autumn vegetation phenology in temperate China. Global Change Biol. 22 (2), 644–655. doi: 10.1111/gcb.13081 26340580

[B43] Liu Q. Fu Y. H. Zhu Z. Liu Y. Liu Z. Huang M.. (2016b). Delayed autumn phenology in the northern hemisphere is related to change in both climate and spring phenology. Global Change Biol. 22 (11), 3702–3711. doi: 10.1111/gcb.13311 27061925

[B44] Lu L. M. Mao L. F. Yang T. Ye J. F. Liu B. Li H. L.. (2018). Evolutionary history of the angiosperm flora of China. Nature 554 (7691), 234–238. doi: 10.1038/nature25485 29420476

[B45] Menzel A. Sparks T. H. Estrella N. Koch E. Aasa A. Ahas R.. (2006). European Phenological response to climate change matches the warming pattern. Global Change Biol. 12 (10), 1969–1976. doi: 10.1111/j.1365-2486.2006.01193.x

[B46] Nagy L. Kreyling J. Gellesch E. Beierkuhnlein C. Jentsch A. (2013). Recurring weather extremes alter the flowering phenology of two common temperate shrubs. Int. J. Biometeorol. 57 (4), 579–588. doi: 10.1007/s00484-012-0585-z 22895652

[B47] Panchen Z. A. Primack R. B. Gallinat A. S. Nordt B. Stevens A. D. Du Y.. (2015). Substantial variation in leaf senescence times among 1360 temperate woody plant species: implications for phenology and ecosystem processes. Ann. Bot. 116 (6), 865–873. doi: 10.1093/aob/mcv015 25808654 PMC4640117

[B48] Pangtey Y. Rawal R. S. Bankoti N. S. Samant S. S. (1990). Phenology of high-altitude plants of kumaun in central himalaya, India. Int. J. Biometeorol. 34 (2), 122–127. doi: 10.1007/BF01093457

[B49] Pau S. Wolkovich E. M. Cook B. I. Davies T. J. Kraft N. J. Bolmgren K.. (2011). Predicting phenology by integrating ecology, evolution and climate science. Global Change Biol. 17 (12), 3633–3643. doi: 10.1111/j.1365-2486.2011.02515.x

[B50] Peñuelas J. Rutishauser T. Filella I. (2009). Phenology feedbacks on climate change. Science 324 (5929), 887–888. doi: 10.1126/science.1173004 19443770

[B51] Piao S. Liu Q. Chen A. Janssens I. A. Fu Y. Dai J.. (2019a). Plant phenology and global climate change: Current progresses and challenges. Global Change Biol. 25 (6), 1922–1940. doi: 10.1111/gcb.14619 30884039

[B52] Piao S. Wang X. Wang K. Li X. Sitch S. (2019b). Interannual variation of terrestrial carbon cycle: Issues and perspectives. Global Change Biol. 26 (1), 300–318. doi: 10.1111/gcb.14884 31670435

[B53] Qian H. Jin Y. (2020). Are phylogenies resolved at the genus level appropriate for studies on phylogenetic structure of species assemblages? Plant Diversity 43 (4), 255–263. doi: 10.1016/j.pld.2020.11.005 34485767 PMC8390917

[B54] Rathcke B. Lacey E. P. (1985). Phenological patterns of terrestrial plants. Annu. Rev. Ecol. Systematics 16, 179–214. doi: 10.1146/annurev.es.16.110185.001143

[B55] Ren P. Liu Z. Zhou X. Peng C. P. Xiao J. Wang S.. (2021). Strong controls of daily minimum temperature on the autumn photosynthetic phenology of subtropical vegetation in China. For. Ecosyst. 8, 31. doi: 10.1186/s40663-021-00309-9 34721934 PMC8550766

[B56] Ren S. Peichl M. (2021). Enhanced spatiotemporal heterogeneity and the climatic and biotic controls of autumn phenology in northern grasslands. Sci. Total Environ. 788, 147806. doi: 10.1016/j.scitotenv.2021.147806 34029811

[B57] Revell L. J. (2011). Phytools: An r package for phylogenetic comparative biology (and other things). Methods Ecol. Evol. 3 (2), 217–223. doi: 10.1111/j.2041-210X.2011.00169.x

[B58] Richardson A. D. Keenan T. F. Migliavacca M. Ryu Y. Sonnentag O. Toomey M. (2013). Climate change, phenology, and phenological control of vegetation feedbacks to the climate system. Agric. For. Meteorol. 169, 156–173. doi: 10.1016/j.agrformet.2012.09.012

[B59] Rosbakh S. Hartig F. Sandanov D. V. Bukharova E. V. Miller T. K. Primack R. B. (2021). Siberian Plants shift their phenology in response to climate change. Global Change Biol. 27 4435–4448. doi: 10.1111/gcb.15744 PMC13420798 34101938

[B60] Schwartz M. D. (2013). Phenology: An integrative environmental science (Dordrecht: Springer Netherlands), 1–5.

[B61] Shen M. Jiang N. Peng D. Rao Y. Tang Y. (2020). Can changes in autumn phenology facilitate earlier green-up date of northern vegetation? Agric. For. Meteorol. 291 (4), 108077. doi: 10.1016/j.agrformet.2020.108077

[B62] Shi C. Sun G. Zhang H. Xiao B. Ze B. Zhang N.. (2014). Effects of warming on chlorophyll degradation and carbohydrate accumulation of alpine herbaceous species during plant senescence on the Tibetan plateau. PloS One 9 (9), e107874. doi: 10.1371/journal.pone.0107874 25232872 PMC4169446

[B63] Sofo A. Dichio B. Montanaro G. Xiloyannis C. (2009). Photosynthetic performance and light response of two olive cultivars under different water and light regimes. Photosynthetica 47 (4), 602–608. doi: 10.1007/s11099-009-0086-4

[B64] Staggemeier V. G. Diniz-Filho J. A. F. Zipparro V. B. Gressler E. de Castro E. R. Mazine F.. (2015). Clade-specific responses regulate phenological patterns in Neotropical myrtaceae. Perspect. Plant Ecol. Evol. Systematics 17 (6), 476–490. doi: 10.1016/j.ppees.2015.07.004

[B65] Vitasse Y. Francois C. Delpierre N. Dufrene E. Kremer A. Chuine I.. (2011). Assessing the effects of climate change on the phenology of European temperate trees. Agric. For. Meteorol. 151, 969–980. doi: 10.1016/j.agrformet.2011.03.003

[B66] Vitasse Y. Porté A. Kremer A. Michalet R. Delzon S. (2009). Responses of canopy duration to temperature changes in four temperate tree species: relative contributions of spring and autumn leaf phenology. Oecologia 161 (1), 187–198. doi: 10.1007/s00442-009-1363-4 19449036

[B67] Wan M. Liu X. (1979). Method of phenology observation of China (Beijing: Science Press), 1–22.

[B68] Wan S. Xia J. Liu W. Niu S. (2009). Photosynthetic overcompensation under nocturnal warming enhances grassland carbon sequestration. Ecology 90 (10), 2700–2710. doi: 10.1890/08-2026.1 19886480

[B69] Wu L. Ma X. Dou X. Zhu J. Zhao C. (2021). Impacts of climate change on vegetation phenology and net primary productivity in arid central Asia. Sci. Total Environ. 796 (8), 149005. doi: 10.1016/j.scitotenv.2021.149055 34328878

[B70] Wu C. Wang J. Ciais P. Peuelas J. Zhang X. Sonnentag O.. (2021). Widespread decline in winds delayed autumn foliar senescence over high latitudes. Proc. Natl. Acad. Sci. 118 (16), e2015821118. doi: 10.1073/pnas.2015821118 33846246 PMC8072329

[B71] Wu C. Wang X. Wang H. Ciais P. Peñuelas J. Myneni R. B.. (2018). Contrasting responses of autumn-leaf senescence to daytime and night-time warming. Nat. Climate Change 9 (2), 177. doi: 10.1038/s41558-018-0346-z

[B72] Xie Y. Wang X. Silander J. A. Jr. (2015). Deciduous forest responses to temperature, precipitation, and drought imply complex climate change impacts. Proc. Natl. Acad. Sci. 112 (44), 13585–13590. doi: 10.1073/pnas.1509991112 26483475 PMC4640769

[B73] Yang Z. Du Y. Shen M. Jiang N. Liang E. Zhu W.. (2021). Phylogenetic conservatism in heat requirement of leaf-out phenology, rather than temperature sensitivity, in Tibetan plateau. Agric. For. Meteorol. 304, 108413. doi: 10.1016/j.agrformet.2021.108413

[B74] Yang K. Jie H. Tang W. Qin J. Cheng C. (2010). On downward shortwave and longwave radiations over high altitude regions: Observation and modeling in the Tibetan plateau. Agric. For. Meteorol. 150 (1), 38–46. doi: 10.1016/j.agrformet.2009.08.004

[B75] Yang Z. Shen M. Jia S. Guo L. Yang W. Wang C.. (2017). Asymmetric responses of the end of growing season to daily maximum and minimum temperatures on the Tibetan plateau. J. Geophys. Res.: Atmospheres 122 (24), 13,278–13,287. doi: 10.1002/2017JD027318

[B76] Yuan M. Wang L. Lin A. Liu Z. Qu S. (2018). Variations in land surface phenology and their response to climate change in Yangtze river basin during 1982–2015. Theor. Appl. Climatol. 137 (3-4), 1659–1674. doi: 10.1007/s00704-018-2699-7

[B77] Zani D. Crowther T. W. Mo L. Renner S. S. Zohner C. M. (2020). Increased growing-season productivity drives earlier autumn leaf senescence in temperate trees. Science 370 (6520), 1066–1071. doi: 10.1126/science.abd8911 33243884

[B78] Zhang X. Liu L. Liu Y. Senthilnath J. Schaaf C. B. (2018). Generation and evaluation of the VIIRS land surface phenology product. Remote Sens. Environ. 216, 212–229. doi: 10.1016/j.rse.2018.06.047

[B79] Zohner C. M. Benito B. M. Fridley J. D. Svenning J. C. Renner S. S. (2017). Spring predictability explains different leaf-out strategies in the woody floras of north America, Europe and East Asia. Ecol. Lett. 20 (4), 452–460. doi: 10.1111/ele.12746 28194867

[B80] Zohner C. ,. M. Benito B. ,. M. Svenning J. ,. C. Renner S. ,. S. (2016). Day length unlikely to constrain climate-driven shifts in leaf-out times of northern woody plants. Nat. Climate Change 6 (12), 1120–1123. doi: 10.1038/nclimate3138

[B81] Zohner C. M. Renner S. S. (2014). Common garden comparison of the leaf-out phenology of woody species from different native climates, combined with herbarium records, forecasts long-term change. Ecol. Lett. 17 (8), 1016–1025. doi: 10.1111/ele.12308 24943497

