# Supplementary material for: Autumn phenology of tree species in China is associated more with climate than with spring phenology and phylogeny
Source: Front Plant Sci. 2023 Jan 19;14:1040758. doi: 10.3389/fpls.2023.1040758 (PMC9893028; doi:10.3389/fpls.2023.1040758)
Supplement: Supplementary file 1 [file DataSheet_1.pdf]

Table S1 Summary of the species studied

No.	Species	Deciduousness	Growth form		NE	N	NW	E	SW
1	*Acer negundo*	d	w	p			√		
2	*Acer pictum subsp. Mono*	d	w	p		√			
3	*Ailanthus altissima*	d	w	p		√	√		
4	*Alangium chinense*	d	w	p					√
5	*Albizia julibrissin*	d	w	p		√	√	√	
6	*Amorpha fruticosa*	d	w	p			√		
7	*Amygdalus davidiana*	d	w	p		√	√		
8	*Amygdalus triloba*	d	w	p	√	√			
9	*Armeniaca mume*	d	w	p					√
10	*Armeniaca sibirica*	d	w	p	√				
11	*Armeniaca vulgaris*	d	w	p		√	√		√
12	*Betula dahurica*	d	w	p	√				
13	*Betula platyphylla*	d	w	p				√	
14	*Bischofia polycarpa*	d	w	p				√	
15	*Bothrocaryum controversum*	d	w	p			√		
16	*Caesalpinia decapetala*	d	w	p					√
17	*Camptotheca acuminata*	d	w	p					√
18	*Caragana arborescens*	d	w	p	√				
19	*Castanea mollissima*	d	w	p		√			
20	*Catalpa bungei*	d	w	p		√			√
21	*Catalpa ovata*	d	w	p	√			√	√
22	*Celastrus flagellaris*	d	w	p	√				
23	*Celtis sinensis*	d	w	p					√
24	*Cerasus pseudocerasus*	d	w	p	√				
25	*Cerasus subhirtella*	d	w	p					√
26	*Cerasus tomentosa*	d	w	p	√				
27	*Cerasus yedoensis*	d	w	p		√			
28	*Cercis chinensis*	d	w	p			√	√	√
29	*Chaenomeles sinensis*	d	w	p			√	√	√
30	*Chaenomeles speciosa*	d	w	p					√
31	*Chimonanthus praecox*	d	w	p			√	√	√
32	*Clerodendrum trichotomum*	d	w	p			√		√
33	*Corylus heterophylla*	d	w	p	√		√		
34	*Cotinus coggygria*	d	w	p		√			
35	*Crataegus pinnatifida*	d	w	p		√			
36	*Crataegus pinnatifida* var. *major*	d	w	p	√				
37	*Diospyros kaki*	d	w	p		√	√	√	
38	*Diospyros lotus*	d	w	p		√			
39	*Elaeagnus angustifolia*	d	w	p			√		
40	*Euonymus alatus*	d	w	p	√				
41	*Firmiana platanifolia*	d	w	p		√	√	√	√

42	*Flueggea suffruticosa*	d	w	p			√		
43	*Forsythia suspensa*	d	w	p		√	√		
44	*Forsythia viridissima*	d	w	p					√
45	*Fraxinus chinensis*	d	w	p		√	√		√
46	*Fraxinus rhynchophylla*	d	w	p	√				
47	*Ginkgo biloba*	d	w	p		√	√	√	√
48	*Gleditsia sinensis*	d	w	p					√
49	*Hedera nepalensis* var. *sinensis*	e	w	p					√
50	*Hibiscus mutabilis*	d	w	p					√
51	*Hibiscus syriacus*	d	w	p		√	√	√	√
52	*Hydrangea macrophylla*	d	w	p		√			
53	*Indigofera kirilowii*	d	w	p	√				
54	*Juglans mandshurica*	d	w	p	√				
55	*Juglans regia*	d	w	p			√		
56	*Kalopanax septemlobus*	d	w	p					√
57	*Koelreuteria bipinnata* var. *integrifoliola*	d	w	p			√		
58	*Koelreuteria paniculata*	d	w	p		√	√		
59	*Lagerstroemia indica*	d	w	p		√	√	√	√
60	*Lespedeza bicolor*	d	w	p	√				
61	*Liquidambar formosana*	d	w	p				√	
62	*Liriodendron chinense*	d	w	p				√	√
63	*Lonicera ferdinandi*	d	w	p	√				
64	*Lonicera maackii*	d	w	p	√				
65	*Lonicera tatarinowii*	d	w	p	√				
66	*Maackia amurensis*	d	w	p	√				
67	*Magnolia denudata*	d	w	p		√	√	√	√
68	*Malus baccata*	d	w	p	√				
69	*Malus micromalus*	d	w	p		√			
70	*Melia azedarach*	d	w	p				√	
71	*Metasequoia glyptostroboides*	d	w	p		√	√		√
72	*Morus alba*	d	w	p	√	√	√		
73	*Padus racemosa*	d	w	p	√				
74	*Paeonia suffruticosa*	d	w	p	√	√	√		
75	*Paulownia fortunei*	d	w	p	√				
76	*Periploca sepium*	d	w	p			√		
77	*Phellodendron amurense*	d	w	p	√				
78	*Philadelphus schrenkii*	d	w	p	√				
79	*Pistacia chinensis*	d	w	p			√		
80	*Platanus occidentalis*	d	w	p				√	
81	*Poncirus trifoliata*	d	w	p			√		
82	*Populus canadensis*	d	w	p		√		√	

83	*Populus davidiana*	d	w	p	√				
84	*Populus euphratica*	d	w	p			√		
85	*Populus pseudosimonii*	d	w	p	√				
86	*Populus simonii*	d	w	p	√				
87	*Populus tomentosa*	d	w	p		√	√		
88	*Populus yunnanensis*	d	w	p					√
89	*Potentilla fruticosa*	d	w	p	√				
90	*Prunus salicina*	d	w	p	√				
91	*Pterocarya stenoptera*	d	w	p			√		√
92	*Punica granatum*	d	w	p		√		√	√
93	*Pyrus pyrifolia*	d	w	p					√
94	*Quercus acutissima*	d	w	p					√
95	*Quercus dentata*	d	w	p	√				
96	*Quercus variabilis*	d	w	p			√		
97	*Rhamnus davurica*	d	w	p	√				
98	*Rhododendron dauricum*	d	w	p	√				
99	*Rhododendron mucronulatum*	d	w	p	√				
100	*Rosa moyesii*	d	w	p			√		
101	*Rosa rugosa*	d	w	p	√				
102	*Rosa xanthina*	d	w	p		√			
103	*Salix babylonica*	d	w	p	√		√	√	√
104	*Salix chaenomeloides*	d	w	p					√
105	*Salix matsudana*	d	w	p	√	√			
106	*Salix matsudana* f. *pendula*	d	w	p		√			
107	*Sapium sebiferum*	d	w	p			√	√	√
108	*Securinega suffruticosa*	d	w	p	√				
109	*Serissa japonica*	d	w	p					√
110	*Sophora japonica* var. *japonica f. pendula*	d	w	p					√
111	*Sorbaria sorbifolia*	d	w	p	√		√		
112	*Spiraea fritschiana*	d	w	p	√				
113	*Spiraea salicifolia*	d	w	p	√				
114	*Spiraea thunbergii*	d	w	p	√				
115	*Spiraea trichocarpa*	d	w	p	√				
116	*Swida paucinervis*	d	w	p					√
117	*Syringa oblata*	d	w	p	√	√	√		
118	*Syringa oblata* var. *alba*	d	w	p		√			
119	*Syringa reticulate* var. *amurensis*	d	w	p	√				
120	*Syringa vulgaris*	d	w	p	√				
121	*Syringa wolfii*	d	w	p	√				
122	*Tamarix laxa*	d	w	p			√		
123	*Tamarix ramosissima*	d	w	p			√		

124	*Tilia amurensis*	d	w	p	√				
125	*Ulmus davidiana* var. *japonica*	d	w	p	√				
126	*Ulmus laevis*	d	w	p				√	
127	*Ulmus pumila*	d	w	p	√	√		√	
128	*Viburnum burejaeticum*	d	w	p	√				
129	*Viburnum opulus* var. *calvescens*	d	w	p	√				
130	*Vitis amurensis*	d	w	p	√				
131	*Vitis vinifera*	d	w	p					√
132	*Weigela florida*	d	w	p	√				
133	*Wisteria sinensis*	d	w	p		√	√	√	√
134	*Xanthoceras sorbifolia*	d	w	p			√		
135	*Ziziphus jujuba*	d	w	p		√			
	Total number of the species				55	37	44	24	40

Note: d: deciduous; e: evergreen w: woody; p: perennial; NE: northeast; N: north; NW: northwest; E: east; SW: southwest.

Table S2. Correlation coefficient between the climate data from meteorological stations and China Meteorological Forcing Dataset (CMFD) at each site.

Sites	Tmax	Tmin	Pre	Win
Beijing	0.99**	0.98**	0.74**	0.84**
Nanchang	0.99**	0.98**	0.77**	0.88**
Hefei	0.99**	0.98**	0.73**	0.87**
Minqin	0.99**	0.98**	0.63**	0.84**
Changchun	0.99**	0.98**	0.73**	0.91**
Mudanjiang	0.99**	0.98**	0.73**	0.89**
Harbin	0.99**	0.97**	0.77**	0.89**

**p<0.01 Tmax: daily maximum temperature, Tmin: daily minimum temperature, Pre: precipitation, Win: wind speed. Note: As China Meteorological Forcing Dataset (CMFD) does not include shortwave radiation data, the result of the correlation between the shortwave radiation from meteorological stations and CMFD is not shown.

Table S3 Information of climate factors in summer and autumn (from June to November) of each site

**Region**	**Site**	Mean Tmax(°C)	Mean Tmin(°C)	Total Pre(mm)	Total Ins(MJ/m^2)	Mean Win(m/s)
Northeast	Harbin	18.27	9.86	448.99	2620.59	2.88
	Mudanjiang	18.20	8.35	446.46	2516.44	2.02
	Changchun	18.88	12.23	480.95	2678.69	2.78
North	Beijing	23.02	15.23	478.49	2743.97	2.93
Northwest	Minqin	21.36	10.49	118.93	3372.62	2.72
	Xi'an	21.99	12.49	380.49	2901.02	2.74
East	Hefei	25.75	19.21	652.42	2594.71	2.52
	Nanchang	27.32	21.68	792.28	2788.36	2.14
Southwest	Chongqing	26.29	24.93	779.79	2189.15	1.52
	Guiyang	23.49	18.05	774.48	2221.43	1.67

Table S4 Native regions of the studied species

No.	Species	Native regions from direct records	Continents with most abundant occurrence records
1	*Acer negundo*	A	
2	*Acer pictum subsp. Mono*	A	
3	*Ailanthus altissima*	A、E	
4	*Alangium chinense*		A
5	*Albizia julibrissin*	A	
6	*Amorpha fruticosa*	NA、SA	
7	*Amygdalus davidiana*		A
8	*Amygdalus triloba*		A
9	*Armeniaca mume*		A
10	*Armeniaca sibirica*		A
11	*Armeniaca vulgaris*		A、E
12	*Betula dahurica*		A、E
13	*Betula platyphylla*		A
14	*Bischofia polycarpa*		A
15	*Bothrocaryum controversum*		A
16	*Caesalpinia decapetala*		A
17	*Camptotheca acuminata*		A
18	*Caragana arborescens*	A	
19	*Castanea mollissima*	A	
20	*Catalpa bungei*		A
21	*Catalpa ovata*	A、E	
22	*Celastrus flagellaris*		A
23	*Celtis sinensis*		A
24	*Cerasus pseudocerasus*		A
25	*Cerasus subhirtella*		A
26	*Cerasus tomentosa*		A

27	*Cerasus yedoensis*		A
28	*Cercis chinensis*		A
29	*Chaenomeles sinensis*		NA、SA
30	*Chaenomeles speciosa*		A
31	*Chimonanthus praecox*		A
32	*Clerodendrum trichotomum*		A
33	*Corylus heterophylla*		A、E
34	*Cotinus coggygria*		E
35	*Crataegus pinnatifida*		A、NA
36	*Crataegus pinnatifida* var. *major*		A、NA
37	*Diospyros kaki*	A	
38	*Diospyros lotus*	A	
39	*Elaeagnus angustifolia*	A、E、NA	
40	*Euonymus alatus*	A、E	
41	*Firmiana platanifolia*		A
42	*Flueggea suffruticosa*		A
43	*Forsythia suspensa*	A、E	
44	*Forsythia viridissima*	A、E	
45	*Fraxinus chinensis*	A	
46	*Fraxinus rhynchophylla*		A
47	*Ginkgo biloba*	A	
48	*Gleditsia sinensis*		A
49	*Hedera nepalensis* var. *sinensis*		A
50	*Hibiscus mutabilis*		A
51	*Hibiscus syriacus*	A	
52	*Hydrangea macrophylla*	A、E	
53	*Indigofera kirilowii*		A
54	*Juglans mandshurica*	A、E	
55	*Juglans regia*	A、E	
56	*Kalopanax septemlobus*		A
57	*Koelreuteria bipinnata* var. *integrifoliola*		A
58	*Koelreuteria paniculata*	A	
59	*Lagerstroemia indica*	A	
60	*Lespedeza bicolor*		A
61	*Liquidambar formosana*	A	
62	*Liriodendron chinense*		A
63	*Lonicera ferdinandi*		A
64	*Lonicera maackii*	A、E	
65	*Lonicera tatarinowii*		A
66	*Maackia amurensis*	A	
67	*Magnolia denudata*	A	
68	*Malus baccata*		A
69	*Malus micromalus*		A

70	*Melia azedarach*	A	
71	*Metasequoia glyptostroboides*	A	
72	*Morus alba*	A	
73	*Padus racemosa*		A
74	*Paeonia suffruticosa*		A
75	*Paulownia fortunei*	A	
76	*Periploca sepium*		A
77	*Phellodendron amurense*		A、E
78	*Philadelphus schrenkii*	A	
79	*Pistacia chinensis*		A
80	*Platanus occidentalis*	NA	
81	*Poncirus trifoliata*	A	
82	*Populus canadensis*	E	
83	*Populus davidiana*		A
84	*Populus euphratica*	A	
85	*Populus pseudosimonii*		A
86	*Populus simonii*	A	
87	*Populus tomentosa*	A	
88	*Populus yunnanensis*	A	
89	*Potentilla fruticosa*		A、E
90	*Prunus salicina*		A
91	*Pterocarya stenoptera*	A	
92	*Punica granatum*	A、E	
93	*Pyrus pyrifolia*		A
94	*Quercus acutissima*		A
95	*Quercus dentata*	A	
96	*Quercus variabilis*		A
97	*Rhamnus davurica*	A	
98	*Rhododendron dauricum*		A
99	*Rhododendron mucronulatum*		A
100	*Rosa moyesii*		A
101	*Rosa rugosa*		E
102	*Rosa xanthina*		A
103	*Salix babylonica*		A、E
104	*Salix chaenomeloides*		A
105	*Salix matsudana*		NA、A
106	*Salix matsudana* f. *pendula*		A
107	*Sapium sebiferum*		A
108	*Securinega suffruticosa*		A
109	*Serissa japonica*		A
110	*Sophora japonica* var. *japonica f. pendula*	A、E	
111	*Sorbaria sorbifolia*	A、E	
112	*Spiraea fritschiana*		A

113	*Spiraea salicifolia*	A、E	
114	*Spiraea thunbergii*		NA、A
115	*Spiraea trichocarpa*		A
116	*Swida paucinervis*		A
117	*Syringa oblata*		A
118	*Syringa oblata* var. *alba*		A
119	*Syringa reticulate* var. *amurensis*		A
120	*Syringa vulgaris*	A、E	
121	*Syringa wolfii*		A
122	*Tamarix laxa*		A
123	*Tamarix ramosissima*	A、E	
124	*Tilia amurensis*		A
125	*Ulmus davidiana* var. *japonica*		A
126	*Ulmus laevis*	E	
127	*Ulmus pumila*	A、NA	
128	*Viburnum burejaeticum*	A	
129	*Viburnum opulus* var. *calvescens*		A
130	*Vitis amurensis*		A
131	*Vitis vinifera*	A、E	
132	*Weigela florida*		A、E
133	*Wisteria sinensis*		NA、E
134	*Xanthoceras sorbifolia*		NA、A
135	*Ziziphus jujuba*	A	
	Total number of the species	53	82

Note: NA: North America; SA: South America; E: Europe; A: Asia.

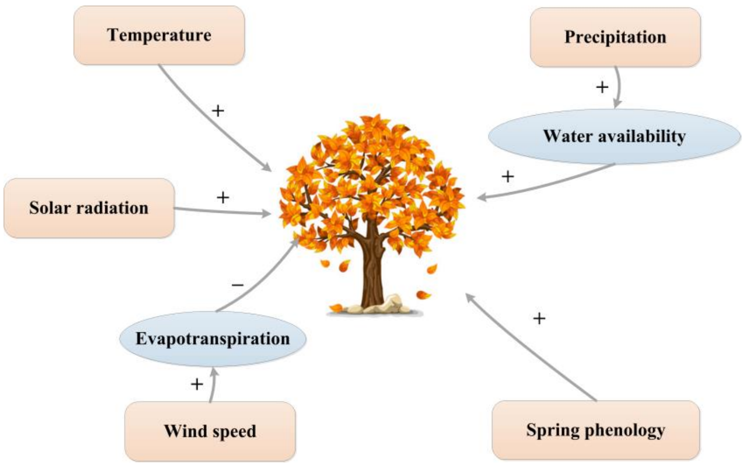

Figure S1. Potential relationships between autumn phenology and climate factors reported in previous studies. The symbol "+" ("–") represents positive (negative) forcing imposed on autumn phenology from each climate factor.

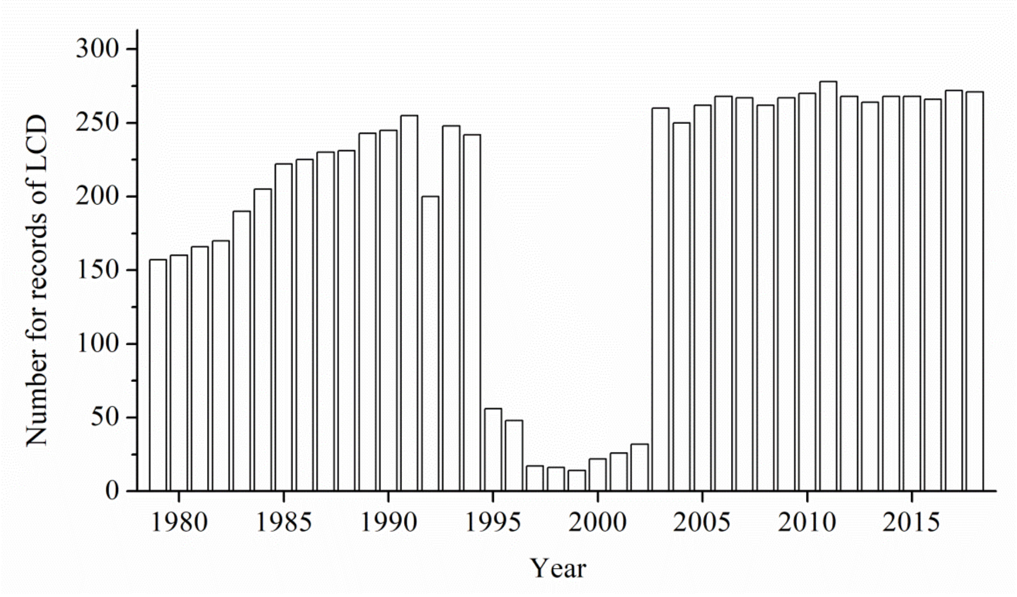

Figure S2. The total number of the observation records for leaf coloring date (LCD) at ten sites in China from 1979 to 2018.

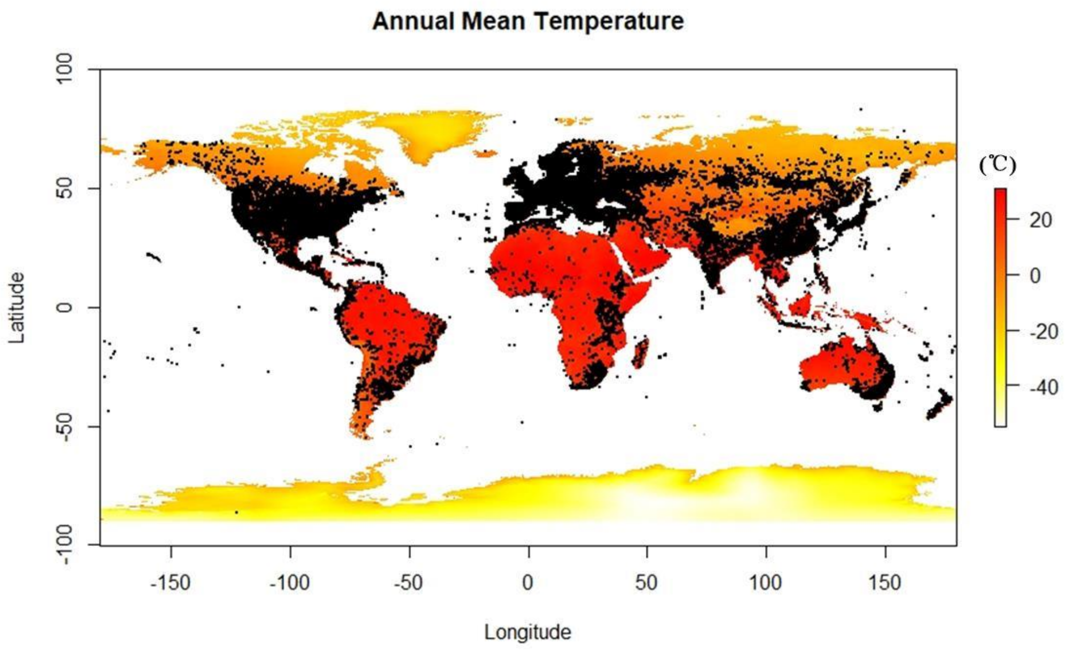

Figure S3. Global distribution of 135 species in this study. The black circles represent the 1421186 occurrence points for all species.
